# Potassium‐Rich Iron Hexacyanoferrate/Carbon Cloth Electrode for Flexible and Wearable Potassium‐Ion Batteries

**DOI:** 10.1002/advs.202305467

**Published:** 2023-12-07

**Authors:** Xinyue Li, Xiaolin Zhang, Junmin Xu, Zhixia Duan, Yue Xu, Xiaosheng Zhang, Lingling Zhang, Ye Wang, Paul K. Chu

**Affiliations:** ^1^ Key Laboratory of Material Physics Ministry of Education School of Physics and Microelectronics Zhengzhou University Zhengzhou 450001 P. R. China; ^2^ Department of Physics Department of Materials Science and Engineering and Department of Biomedical Engineering City University of Hong Kong Tat Chee Avenue Kowloon Hong Kong 999077 P. R. China; ^3^ School of Materials Science and Engineering Zhengzhou University Zhengzhou 450001 P. R. China; ^4^ Department of Chemical and Biological Engineering The Hong Kong University of Science and Technology Clear Water Bay Kowloon Hong Kong 999077 P. R. China

**Keywords:** flexible cathode, good cycling stability, high capacity, K‐rich iron hexacyanoferrate/carbon cloth, wearable potassium ion batteries

## Abstract

The fast development of flexible and wearable electronics increases the demand for flexible secondary batteries, and the emerging high‐performance K‐ion batteries (KIBs) have shown immense promise for the flexible electronics due to the abundant and cost‐effective potassium resources. However, the implementation of flexible cathodes for KIBs is hampered by the critical issues of low capacity, rapid capacity decay with cycles, and limited initial Coulombic efficiency. To address these pressing issues, a freestanding K‐rich iron hexacyanoferrate/carbon cloth (KFeHCF/CC) electrode is designed and fabricated by cathodic deposition. This innovative binder‐free and self‐supporting KFeHCF/CC electrode not only provides continuous conductive channels for electrons, but also accelerates the diffusion of potassium ions through the active electrode–electrolyte interface. Moreover, the nanosized potassium iron hexacyanoferrate particles limit particle fracture and pulverization to preserve the structure and stability during cycling. As a result, the K‐rich KFeHCF/CC electrode shows a reversible discharging capacity of 110.1 mAh g^−1^ at 50 mA g^−1^ after 100 cycles in conjunction with capacity retention of 92.3% after 1000 cycles at 500 mA g^−1^. To demonstrate the commercial feasibility, a flexible tubular KIB is assembled with the K‐rich KFeHCF/CC electrode, and excellent flexibility, capacity, and stability are observed.

## Graphical abstract

In this study, a flexible and freestanding K‐rich KFeHCF/CC electrode is prepared by cathodic deposition to form the cathode in a flexible K‐ion battery (KIB). The flexible and wearable tubular KIB constructed with the potassium‐rich iron hexacyanoferrate cathode shows excellent flexibility, high capacity, and good stability. The results show that the electrode materials have a large potential in flexible potassium‐ion energy storage devices.

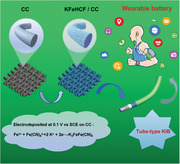

## 1 Introduction

Among the emerging electrochemical power systems, K‐ion batteries (KIBs) have garnered great interests due to the cost‐effectiveness in terms of raw materials, low standard redox potential of K^+^/K, and mature graphite anode.^[^ ^1^ ^]^ For instance, the standard electrode potential versus the standard hydrogen electrode (SHE) of K^+^/K (−2.93 V) is in between Li^+^/Li (−3.04 V vs SHE) and Na^+^/Na (−2.71 V vs SHE) couples. However, the theoretical and practical calculations demonstrate that the K^+^/K redox couple could exhibit lowest reduction potential (−2.88 V vs SHE) in commonly used carbonate electrolyte like propylene carbonate (PC), as compared to Li^+^/Li (−2.79 V vs SHE) and Na^+^/Na (−2.56 V vs SHE).^[^ ^1^ ^]^ This could result in a wider potential window and high energy density for KIBs. Another notable advantage of K‐ion batteries, compared to Li‐ion batteries and Na‐ion batteries, is the much weaker Lewis acidity of K^+^ ion, resulting in smaller solvated ions as compared to Li^+^ and Na^+^ ions. The smaller solvated ions and the low desolvation energy could guarantee a faster diffusion through the electrolyte/electrode interface. But, the development of the KIBs is plagued by the fast capacity decay and inferior rate capability due to the large volume change of the host materials as well as sluggish diffusion kinetics during charging/discharging process because of the large radius of K^+^.^[^ ^2^ ^]^ Therefore, it is important to design cathode materials with good structural stability and enhanced diffusion kinetics. In addition, owing to the low cost and natural abundance of potassium, KIBs are expected to play an important role in flexible and wearable electronics.

Several K‐intercalating compounds have been reported as potential cathode materials for KIBs, for example, layered transition metal oxides,^[^ ^3^ ^]^ polyanionic compounds,^[^ ^4^ ^]^ organic compounds,^[^ ^5^ ^]^ as well as Prussian blue and its analogs (PBAs).^[^ ^6^ ^]^ PBAs such as A_2−x_M[Fe(CN)_6_]_1−y_·zH_2_O are hexacyanoferrates in which M represents a transition metal such as Fe, Co, Mn, Cu, Ni, Zn, and so on.^[^ ^7^ ^]^ In particular, potassium iron hexacyanoferrates (KFeHCF), i.e., K_2_Fe^II^Fe^II^(CN)_6_·nH_2_O, attract plenty of interests due to the advantages of low‐cost raw material, high working voltage, and high theoretical capacity of 155.52 mAh g^−1^ contributed from the two redox active sites of low‐spin FeC_6_ and high‐spin FeN_6_ octahedra for the extraction/insertion of two K^+^ ions.^[^ ^8^ ^]^ Additionally, PBAs exhibit a clear preference for K^+^ ion,^[^ ^1a^ ^]^ as to their robust open frameworks for low‐strain K^+^ storage and the appropriate ionic radius of K^+^ ion stabilizes the structure, which results in a better cycling performance than Li‐ion batteries and Na‐ion batteries. Nazar et al. have reported a citrate‐assisted co‐precipitation method to synthesize K_1.69_Fe[Fe(CN)_6_]_0.90_·0.4H_2_O nanoparticles that show a discharging capacity of 120 mAh g^−1^ at a current density of 100 mA g^−1^.^[^ ^9^ ^]^ Komaba et al. have designed a convenient precipitation method to synthesize K_1.64_Fe[Fe(CN)_6_]_0.89_·0.15H_2_O,^[^ ^10^ ^]^ and the materials show a discharging capacity of 130 mAh g^−1^ at 30 mA g^−1^. Chong et al. have demonstrated a one‐step hydrothermal method to synthesize K_2_Ni_x_Fe_1−x_[Fe(CN)_6_]_2_ (0 ≤ x ≤ 1). The K_1.90_Ni_0.5_Fe_0.5_[Fe(CN)_6_]_0.89_·0.42H_2_O sample delivers stable cycling performance with capacity retention of 87.1% after 500 cycles at 20 mA g^−1^.^[^ ^6b^ ^]^

The electrochemical properties of potassium iron hexacyanoferrates and K^+^ storage depend on the composition of the materials which can be tailored by the specific synthetic methods.^[^ ^11^ ^]^ For instance, in electrodeposition, low‐valence (metallic) or high‐valence materials can be produced controllably by electro‐reduction or electro‐oxidation. The electrodeposition parameters such as the applied voltage, current density, solution composition, pH, and temperature can be adjusted to optimize the physical and chemical properties of the materials. Therefore, electrodeposition is widely used to prepare nanocomposites on commercial carbon cloth (CC) which has the merits of high electrical conductivity, good corrosion resistance, and high mechanical strength boding well for flexible electrodes.^[^ ^12^ ^]^ However, up to now, the preparation of potassium‐rich iron hexacyanoferrate on CC as a binder‐free cathode for KIBs has not been reported.

Herein, a freestanding K‐rich iron hexacyanoferrate/carbon cloth (KFeHCF/CC) electrode is prepared by cathodic deposition. The binder‐free and self‐supporting KFeHCF/CC electrode not only provides continuous conductive channels for electrons, but also facilitates rapid diffusion of potassium ions through the active electrode–electrolyte interface. The nanosized potassium iron hexacyanoferrate particles inhibit particle fracture and pulverization, consequently preserving the integrity and stability of the electrode during cycling and giving rise to impressive electrochemical characteristics such as reversible discharge capacity of 110.1 mAh g^−1^ at 50 mA g^−1^ after 100 cycles, capacity retention of 92.3% for 1000 cycles at 500 mA g^−1^, as well as good rate performance of 67.8 mAh g^−1^ at 1500 mA g^−1^. To demonstrate the practical viability, a flexible and wearable tubular KIB is assembled with the K‐rich KFeHCF/CC electrode, and excellent flexibility, capacity, and stability are obtained.

## 2 Results and Discussion

**Figure 1:**
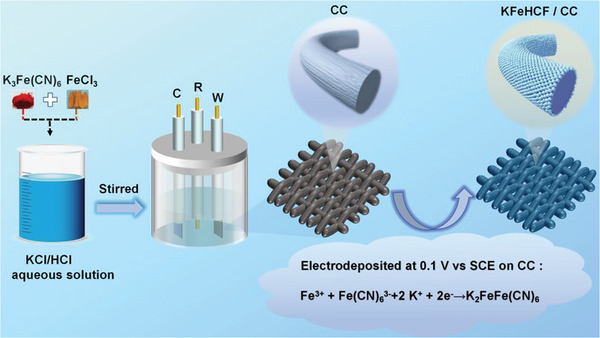
Schematic illustration of the synthesis and formation mechanism of KFeHCF/CC by electrodeposition.

The preparation procedure of the KFeHCF deposited on CC is illustrated by **Figure** **1**. The KFeHCF nanoparticles can uniformed grow on CC when the electrodeposition voltage is set to 0.1 V versus saturated calomel electrode (SCE), which is determined through the CV curve of the pristine synthesis solution at 10 mV s^−1^ scanning from −0.3 to 0.7 V (Figure S1, Supporting Information). In this CV curve, the reduction peak at ∼0.1 V is correlated with the K‐rich KFeHCF growing with simultaneous intercalation of K^+^. The related oxidation peak at ∼0.25 V is assigned to the deintercalation of K^+^ and consequent formation of Prussian blue FeFe(CN)_6_. The relevant electrochemical processes are represented by the following equations:

(1) $${\mathrm{Fe}}^{3+}+\mathrm{Fe}{\left(\mathrm{CN}\right)}_{6}^{3-}+2K^{+}+2e^{-}\to K_{2}\mathrm{FeFe}{\left(\mathrm{CN}\right)}_{6}$$

(2) $$K_{2}\mathrm{FeFe}{\left(\mathrm{CN}\right)}_{6}-2e^{-}\to \mathrm{FeFe}{\left(\mathrm{CN}\right)}_{6}+2K^{+}$$

**Figure 2:**
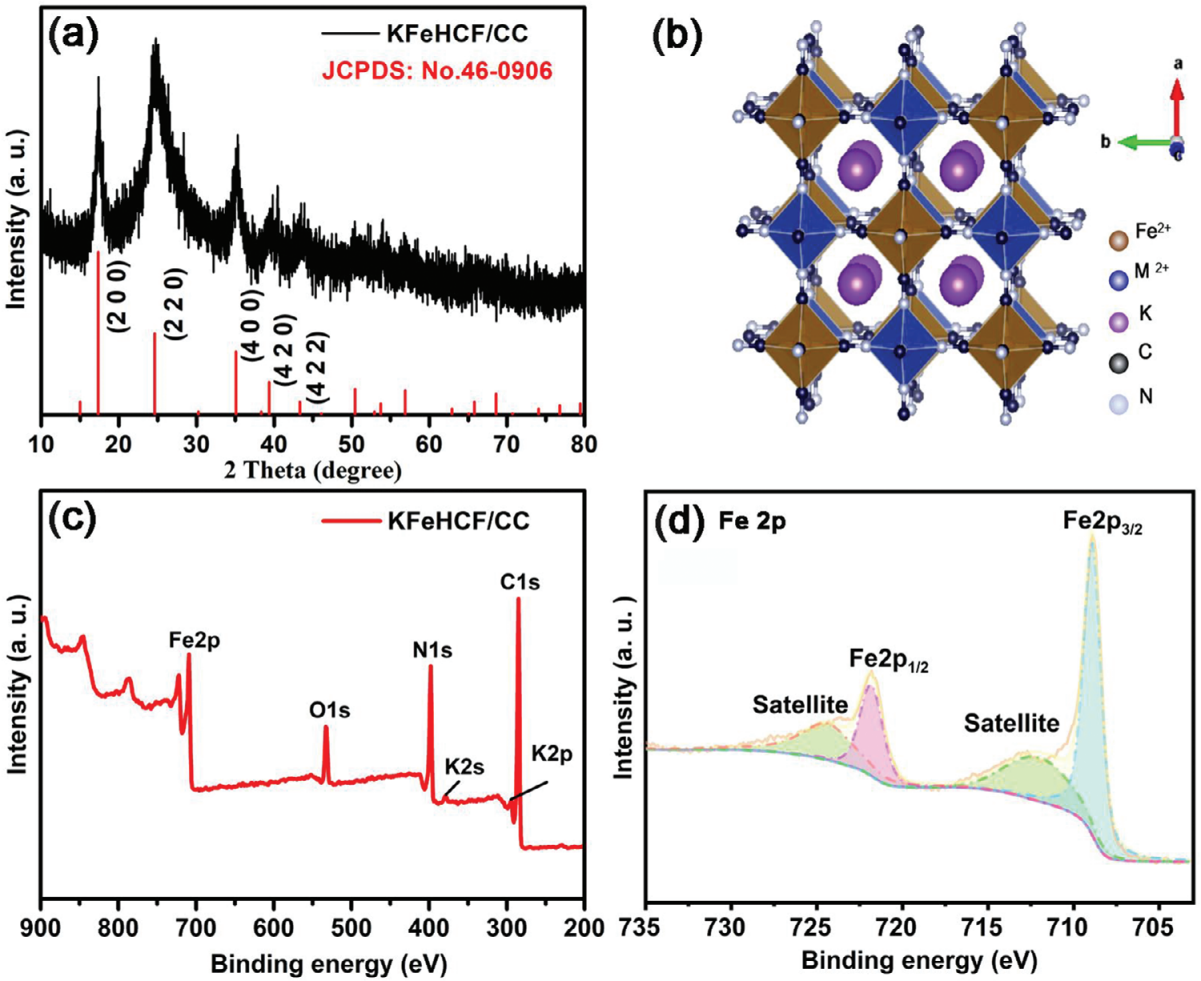
a) XRD patterns of KFeHCF/CC; b) Crystal structure of the face‐centered cubic K_2_MFe(CN)_6_ (M = Fe, Mn, Ni, Co); c) XPS survey spectrum, and d) Fitted Fe 2*p* spectrum of KFeHCF/CC.

Nanosized KFeHCF particles formed uniformly on CC under optimal electrodeposition conditions are first characterized by X‐ray diffraction (XRD). XRD pattern confirms the formation of potassium iron hexacyanoferrates on the carbon cloth, as shown in **Figure** **2**a. The peaks at 2*θ* angles of 17.3, 24.6, 35.2, 39.6, and 43.7, respectively, correspond to (2 0 0), (2 2 0), (4 0 0), (4 2 0), and (4 2 2) planes of the face‐centered cubic (FCC) potassium iron hexacyanoferrates hydrate (JCPDS No. 46‐0906),^[^ ^6d^ ^]^ which indicates high‐purity K_2_FeFe(CN)_6_ is synthesized on CC. Figure 2b exhibits the schematic diagram of the crystal structure of the FCC K_2_M[Fe(CN)_6_](M = Fe, Mn, Ni, Co). In this crystal structure, the Fe^2+^ and M^2+^ ions are alternately sixfold coordinated to the C and N ends of the CN‐ ligand, respectively, forming a 3D open framework with a large interstitial space to accommodate K ions and H_2_O molecules.^[^ ^13^ ^]^

**Figure 3:**
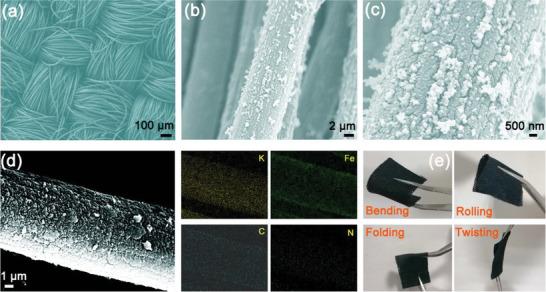
a) Low‐magnification and b,c) High‐magnification SEM images of the as‐prepared KFeHCF/CC, d) SEM image and elemental maps of a single KFeHCF/CC fiber, and e) Optical images of KFeHCF/CC under different bending conditions.

The composition and chemical states are determined by X‐ray photoelectron spectroscopy (XPS). K, Fe, C, N, and O are observed from KFeHCF/CC (Figure 2c). Figure 2d shows two peaks at 708.9 and 721.8 eV corresponding to Fe 2*p*_3/2_ and Fe 2*p*_1/2_, respectively and those at 712.8 and 724.2 eV are the satellites peak.^[^ ^6^, ^14^ ^]^ The results show that bivalent Fe atoms occupy different sites in KFeHCF/CC. One Fe atom occupies the nitrogen‐coordinated sites as high‐spin Fe^II^, forming Fe^II^N_6_ octahedra, while the other Fe atom occupies the carbon‐coordinated sites as low‐spin Fe^II^, forming Fe^II^C_6_ octahedra.^[^ ^6^, ^14^ ^]^ **Figure** **3**a depicts the SEM image of KFeHCF/CC revealing complete and uniform coverage of potassium iron hexacyanoferrate on CC. The high‐magnification SEM images (Figure 3b,c; Figure S3a,b, Supporting Information) show the potassium iron hexacyanoferrate nanoparticles have an average size of 200 nm and attach to the conductive carbon cloth. Figure 3d shows the SEM image of a single carbon fiber and elemental maps confirming that K, Fe, C, and N are distributed uniformly. Figure 3e shows that KFeHCF/CC has high flexibility under different conditions including rolling, twisting, and bending and no obvious cracks are observed after mechanical deformation (Figure S5, Supporting Information). Furthermore, the freestanding and flexible KFeHCF/CC electrode can be made into a custom shape to cater to specific applications such as the cathode in KIBs. Moreover, the freestanding and flexible KFeHCF/CC electrode is also light because neither a metal current collector nor nonconductive binder is needed.

**Figure 4:**
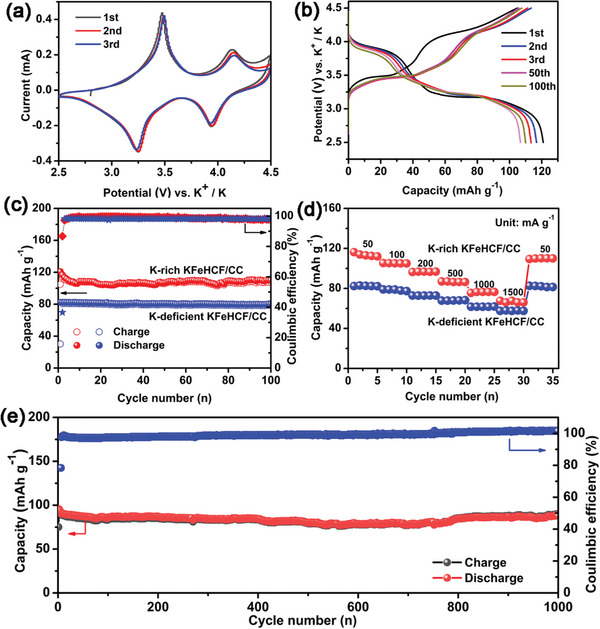
a) Initial CV curves of KFeHCF/CC at a scanning rate of 0.1 mV s^−1^; b) 1st, 2nd, 3rd, 50th, and 100th cycle charging/discharging curves of KFeHCF/CC at 50 mA g^−1^; Electrochemical properties of KFeHCF/CC: c) Cycling characteristics, d) Rate capability, and e) Long‐term cycling stability of the K‐rich KFeHCF/CC electrode at 500 mA g^−1^.

The electrochemical properties of the KFeHCF/CC electrode are evaluated using a coin‐type KIB half‐cell with a potassium metal anode. **Figure** **4**a shows the first three cyclic voltammetry (CV) cycles between 2.5 and 4.5 V at 0.1 mV s^−1^. The two pair of symmetrical redox peaks at 3.21 V/3.52 V and 3.91 V/4.16 V arise from reduction/oxidation of high‐spin Fe^III^/Fe^II^ coordinated with N atoms and low‐spin Fe^III^/Fe^II^ coordinated with C atoms, respectively.^[^ ^6b^ ^]^ The CV curves show good reproducibility indicating a highly reversible K^+^ extraction/insertion behavior during initial cycling. Figure 4b shows the charging/discharging curves of the 1st, 2nd, 3rd, 50th, and 100th cycles at 50 mA g^−1^. The two charge voltage plateaus at 3.50 and 4.10 V are attributed to oxidation of high‐spin Fe^II^ and low‐spin Fe^II^, respectively. Correspondingly, there are two voltage plateaus at 3.20 and 3.90 V in the discharging curves. The initial charging/discharging curves are similar to those of the following cycles, further confirming reversible K storage during long‐term cycling. The 1st cycle charging/discharging capacity is 104.8 mAh g^−1^/120.9 mAh g^−1^ with a high initial Coulombic efficiency of 86.7%. In the subsequent cycles, the reversible capacity is retained and the discharging capacity is maintained at 110.1 mAh g^−1^ after 100 cycles (Figure 4c). For comparison, the electrochemical properties of KFeHCF/CC prepared at 0.4 V (vs SCE) can be seen in Figure 4c. The first cycle charging and discharging capacities are 30.2 and 82.5 mAh g^−1^, respectively and the initial Coulombic efficiency is only 36.6% reflecting inferior performance when the K concentration is small. Although the K‐deficient KFeHCF/CC electrode exhibits high capacity retention rate of 96.8%, the discharging capacity is only 79.9 mAh g^−1^ after 100 cycles. Furthermore, the discharging capacity of K‐rich KFeHCF/CC is higher than that of K‐deficient KFeHCF/CC during cycling due to the large K concentration and fewer structural defects.

The rate performance is another important factor and as shown in Figure 4d, the K‐rich KFeHCF/CC electrode has discharging capacities of 113.5, 104.8, 97.4, 86.3, and 76.8 mAh g^−1^ at current densities of 50, 100, 200, 500, and 1000 mA g^−1^, respectively. Even at a very large current density of 1500 mA g^−1^, the discharging capacity is 67.8 mAh g^−1^ which is 59.7% of the initial reversible capacity. When the current density is reverted back to 50 mA g^−1^, the reversible capacity recovers to 109.6 mAh g^−1^, implying a full recovery of the rate capacity. On the contrary, the K‐deficient KFeHCF/CC electrode shows discharging capacities of 82.7, 79.2, 72.8, 67.7, 63.1, and 57.8 mAh g^−1^ at current densities of 50, 100, 200, 500, 1000, and 1500 mA g^−1^, respectively. The above results demonstrate that the K‐rich KFeHCF/CC electrode has excellent K storage characteristics including high reversible capacities and cycling stability.

**Figure 5:**
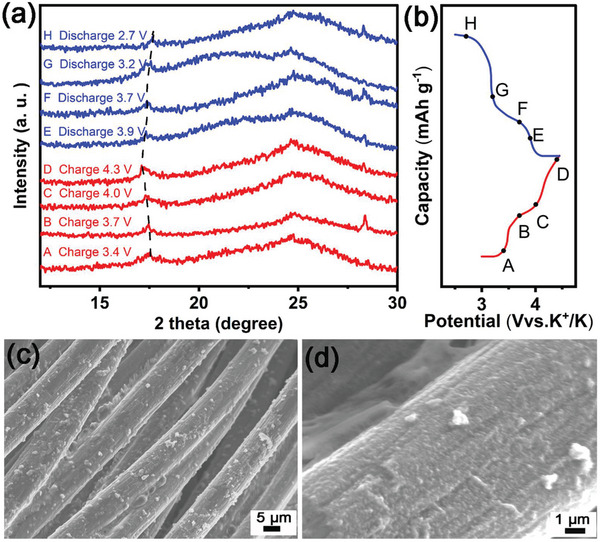
a) Ex situ XRD patterns of KFeHCF/CC in the 1st cycle and b) the corresponding charging/discharging curve and c,d) SEM images of the KFeHCF/CC electrode after 1000 cycles at 500 mA g^−1^.

The long‐cycling characteristics are investigated at a large current density of 500 mA g^−1^. As shown in Figure 4e, a high reversible capacity of 88.3 mAh g^−1^ and 92.3% capacity retention are observed after 1000 cycles at 500 mA g^−1^, which corresponds to an extremely small capacity decay of 0.0077% per cycle. Prussian blue and its analogs with high capacities and long cycling stability have rarely been reported (**Table** **1**). The reason for the good cycling stability of KFeHCF/CC is studied by ex situ XRD. As shown in **Figure** **5**a,b, when the electrode is charged from 3.0 to 4.5 V, the FCC structure is maintained with XRD diffraction peaks shift slightly to smaller angles, which demonstrates tiny cell expansion occurs during the charging process. When the electrode is discharged from 4.5 to 2.5 V, the reversible XRD peaks shifting back can be observed from KFeHCF/CC during K intercalation suggesting the good structural stability for K‐ion storage. More importantly, no structural phase transition during the charging/discharging process confirms the solid solution K insertion reactions for KFeHCF/CC electrode. This can prove that KFeHCF exhibits a clear preference for K^+^ ion, as to their robust open frameworks for low‐strain K^+^ storage, which could lead to a decent cycling performance. Moreover, as shown in Figure 5c,d, the morphology of the KFeHCF/CC electrode after 1000 charging/discharging cycles at 500 mA g^−1^ is almost unchanged. It is because the nanosized KFeHCF particles inhibit fracture and pulverization, as evidenced by that no pulverization and delamination from CC is observed after long cycling. Meanwhile, the carbon cloth provides a flexible substrate to accommodate the volume change during charging/discharging process, consequently enhancing the microstructural stability during cycling.

**Table 1:** Comparison of the cycling stability of our electrode with that of similar electrode materials.

Materials	Operating voltage [V]	Initial discharge capacity [mAh g^−1^]	Capacity retention	Ref.
KFeHCF/CC (this work)	2.5 – 4.5	95.8 at 500 mA g^−1^	92.3 % (1000 cycles)	
K_1.90_Ni_0.5_Fe_0.5_[Fe(CN)_6_]_0.89_·0.42H_2_O	2.0 – 4.5	72.3 at 100 mA g^−1^	82.3 %(1000 cycles)	[6b]
K_0.220_ Fe[Fe(CN)_6_ ] _0.805_·4.01H_2_O	2.0 – 4.0	51.7 at 300 mA g^−1^	86.5 % (300 cycles)	[15a]
KFe[Fe(CN)_6_ ] _0.82_ ·2.87H_2_O	2.0 – 4.5	90.7 at 100 mA g^−1^	80.5 %(1000 cycles)	[6d]
K _1.7_ Fe[Fe(CN)_6_ ] ∙0.9 H_2_O	2.0 – 4.5	120.0 at 100 mA g^−1^	65.0 % (300 cycles)	[9]
K_1.84_Ni[Fe(CN)_6_]_0.88_∙0.49H_2_O	2.0 – 4.5	62.8 at 500 mA g^−1^	88.6 % (100 cycles)	[7a]
K_1.81_Ni[Fe(CN)_6_]_0.97_∙0.086H_2_O	2.0 – 4.5	40.3 at 50 mA g^−1^	87.3 % (1000 cycles)	[7b]

**Figure 6:**
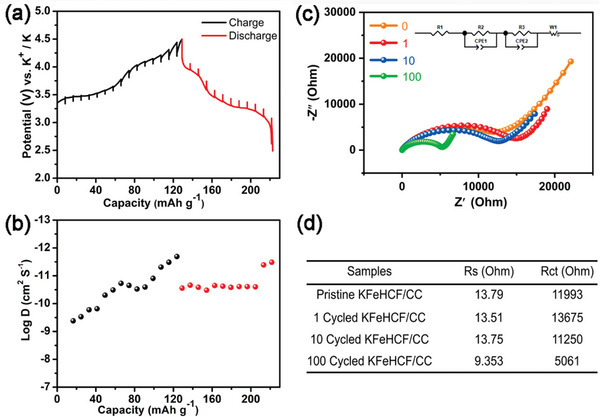
a) GITT profile and b) Calculated diffusion coefficients of the KFeHCF/CC electrode; c) Electrochemical impedance spectra of the electrodes before and after 1, 10, and 100 cycles; d) Fitted Rs and Rct values.

The kinetics is studied by the galvanostatic intermittent titration technique (GITT). **Figure** **6**a,b presents the GITT curves for the second cycle revealing K^+^ diffusion coefficients between 10^−11^ and 10^−9^ cm^2^ s^−1^ during charging/discharging process, which are larger than those of most reported KIB cathodes,^[^ ^3^, ^15^ ^]^ demonstrating the fast potassium ions diffusion kinetics. Electrochemical impedance spectroscopy (EIS) and the corresponding fitting results (Figure 6c,d) reveal that the charge transfer resistance (Rct) decreases gradually from 11 993 to 5061 Ω throughout 100 cycles and it is beneficial to the electrochemical performance. The Rct obviously decreases after long cycling can be attributed to the electrochemical activation and a microstructural superiority of KFeHCF/CC electrode. In the structure, CC provides a large surface area and abundant sites to anchor the KFeHCF nanoparticles and improve electron transfer. At the same time, the absence of a non‐conductive binder avoids an inactive interface that can impede electron transfer and potassium ion transport consequently synergistically improving the electrochemical characteristics.

**Figure 7:**
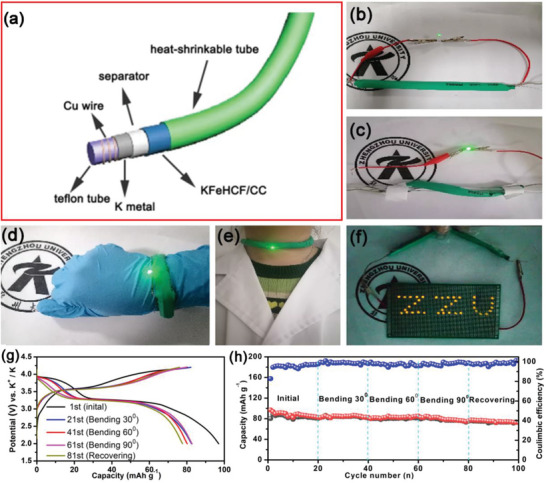
a) Schematic illustration for the structure of the flexible tubular KIB; b–e) LED lit by the KIB under different deformation conditions; f) Powering 38 LEDs; g) Charging/discharging profiles of the flexible KIB at a current density of 50 mA g^−1^; h) Cycling stability of the tubular KIB at 50 mA g^−1^ under different bending conditions.

To demonstrate the commercial viability, the K‐rich KFeHCF/CC electrode is incorporated into a flexible tubular KIB (**Figure** **7**a) to power a red light‐emitting diode (LED) (Figure 7b). More importantly, even under mechanical deformation, it continues to work (Figure 7c–e) and power 38 LEDs in parallel for several hours (Figure 7f). Figure 7g shows the charging/discharging curves at the 1st, 21st, 41st, 61st, and 81st cycles of the tubular KIB at a current density of 50 mA g^−1^. The 1st cycle charging/discharging capacity is 80.3 mAh g^−1^/96.9 mAh g^−1^ with a high Coulombic efficiency of 82.9%. The charging/discharging curves show two charging plateaus and two discharging plateaus, revealing a two‐electron reaction with high capacity at different bending states. Figure 7h shows the cycling characteristics of the KIB at a current density of 50 mA g^−1^. The capacity is almost unchanged after bending to angles of 30^0^ and 90^0^. Meanwhile, the reversible capacity of the flexible device is nearly constant at 72.4 mAh g^−1^ and 74.7% retention is observed after 100 cycles on account of the excellent structural durability and mechanical flexibility during K‐ion intercalation and deintercalation. The good stability stems from the strong adhesion between the KFeHCF nanoparticles and carbon cloth. Hence, delamination can be avoided in spite of severe mechanical deformation. The results suggest that the free‐standing K‐rich KFeHCF/CC electrode has enormous potential for flexible devices.

## 3 Conclusion

A flexible and freestanding K‐rich KFeHCF/CC electrode prepared by cathodic deposition forms the cathode in a K‐ion battery. Owing to the short electron/ion transport path, good structural stability, and high reactivity of K^+^, the K‐rich KFeHCF/CC electrode shows a high reversible charge capacity of 110.1 mAh g^−1^ at 50 mA g^−1^, high rate capability, as well as excellent stability during cycling. The flexible and wearable tubular KIB constructed with the potassium‐rich iron hexacyanoferrate cathode shows excellent flexibility, high capacity, and good stability. The results show that the electrode materials have a large potential in flexible potassium‐ion energy storage devices.

## 4 Experimental Section

### Synthesis of KFeHCF/CC

The K‐rich iron hexacyanoferrate was deposited on carbon cloth (CC) by cathodic deposition using a standard three‐electrode configuration in which the electrolyte consisted of 2.5 mm K_3_Fe(CN)_6_, 2.5 mm FeCl_3_, 1.0 m KCl, and 5.0 mm HCl, a piece of CC (3 cm × 3 cm) was the working electrode, a platinum sheet was the counter electrode, and a saturated calomel electrode (SCE) was the reference electrode. Cathodic deposition was performed at a constant potential of 0.1 V (vs SCE) for 1200 s at room temperature. For comparison, a K‐deficient KFeHCF/CC electrode was also prepared in the same way but at a cathodic deposition potential of 0.4 V. The average mass loading of KFeHCF on CC was ∼2 mg cm^−2^ when the deposition time was set to 1200 s. The average mass of the carbon cloth (W1S1009, CeTech) used in this experiment was ∼14 mg cm^−2^. The mass ratio between the KFeHCF and CC was ∼1:7.

### Materials Characterization

The phases in the samples were investigated by X‐ray diffraction (XRD, PANalytical) with monochromatic Cu K_α_ radiation. The chemical states and composition were determined by X‐ray photoelectron spectroscopy (XPS, ESCALAB 250) and energy‐dispersive X‐ray spectroscopy (EDS). The water contents in the KFeHCF were determined by TG‐DSC (STA449F3, NETZSCH, Germany), where the temperature range was set to be 35–500 °C with a heating rate of 10 °C min^−1^ in N_2_ atmosphere. The morphology and microstructure were examined by field‐emission scanning electron microscopy (FE‐SEM, JSM‐6700F) and transmission electron microscopy (TEM, JEM‐2100).

### Electrochemical Measurements

The mass loading of KFeHCF on the carbon cloth substrate was ≈2 mg cm^−2^ determined by a microbalance (Model‐CPA225D with a resolution of 0.01 mg). The KFeHCF/CC disk with a diameter of 1 cm was incorporated into a CR2032 coin‐type cell as the cathode in an Ar‐filled glove box. A potassium metal sheet and Whatman glass microfiber filter (GF/D) were the anode and separator, respectively, and 2.5 m potassium bis(fluorosulfonyl) imide (KFSI) in triethyl phosphate (TEP) was the electrolyte. The electrochemical properties were determined on the CHI660E electrochemical workstation (Chenhua, Shanghai) by cyclic voltammetry (CVs) and the galvanostatic charging/discharging properties were determined on the Neware battery‐testing instrument. The GITT data were collected at a current density of 50 mA g^−1^ for 300 s, and then followed by a rest of 300 s. The potential was approximate to linearly related to τ^1/2^ (Figure S6, Supporting Information). According to the GITT analytical theory, the potassium ion diffusion coefficient of KFeHCF can be calculated by the following equation:^[^ ^16^ ^]^

(3) $$D=\frac{4}{\pi \tau }{\left(\frac{m_{b}V_{M}}{M_{B}S}\right)}^{2}{\left(\frac{\Delta E_{s}}{\Delta E_{\tau }}\right)}^{2}\;\tau <<\frac{L^{2}}{D}$$

where *D*, *m*_b_, *V*_M_, *M*_B_, and *S* are the diffusion coefficient, active material mass, molar volume of KFeHCF, molar mass of KFeHCF, and area of the electrode, respectively. τ is the current pulse time, L is the average thickness of the KFeHCF. Figure S7 (Supporting Information) shows the ∆*E*τ and ∆*E*s.

A tubular KIB was assembled into a shrinkable plastic tube with KFeHCF/CC and a potassium foil as the cathode and anode, respectively. A Cu wire was wound around the shrinkable plastic tube to form the current collector and the potassium anode, separator, and KFeHCF/CC cathode were wound on the Al wire in a glove box. An aluminum wire was used to wind the positive electrode. Finally, another plastic straw was put in and the electrolyte was added dropwise before sealing.

## Conflict of Interest

The authors declare no conflict of interest.

## Supporting information

Supporting Information

## Data Availability

The data that support the findings of this study are available in the supplementary material of this article.
