# Supplementary material for: Potassium‐Rich Iron Hexacyanoferrate/Carbon Cloth Electrode for Flexible and Wearable Potassium‐Ion Batteries
Source: Adv Sci (Weinh). 2023 Dec 7;11(5):2305467. doi: 10.1002/advs.202305467 (PMC10837388; doi:10.1002/advs.202305467)
Supplement: Supplementary file 1 — Supporting Information [file ADVS-11-2305467-s001.pdf]

ADVANCED SCIENCE
Open Access

Supporting Information

for *Adv. Sci.*, DOI 10.1002/advs.202305467

Potassium-Rich Iron Hexacyanoferrate/Carbon Cloth Electrode for Flexible and Wearable Potassium-Ion Batteries

Xinyue Li, Xiaolin Zhang, Junmin Xu, Zhixia Duan, Yue Xu, Xiaosheng Zhang, Lingling Zhang, Ye Wang* and Paul K. Chu**

Supporting Information

Potassium-rich iron hexacyanoferrate/carbon cloth electrode for flexible and wearable potassium - ion batteries

Xinyue Li, Xiaolin Zhang, Junmin Xu[*]*, Zhixia Duan, Yue Xu, Xiaosheng Zhang,*

Lingling Zhang, Ye Wang[*] *and Paul K. Chu*[*]

* Corresponding author.

X.Li, J. Xu, Z. Duan, Y. Wang

Key Laboratory of Material Physics, Ministry of Education, School of Physics and Microelectronics, Zhengzhou University, Zhengzhou 450001, wP. R. China,

Email: junminxu@zzu.edu.cn (J. Xu);

wangye@zzu.edu.cn (Y. Wang);

X.Zhang, Y.Xu, P.K.Chu

Department of Physics, Department of Materials Science and Engineering, and Department of Biomedical Engineering, City University of Hong Kong, Tat Chee Avenue, Kowloon, Hong Kong, China

Email:paul.chu@cityu.edu.hk (P.K. Chu);

X. Zhang, L. Zhang

School of Materials Science and Engineering, Zhengzhou University, Zhengzhou, 450001, P. R. China

L. Zhang

Department of Chemical and Biological Engineering, The Hong Kong University of Science and Technology, Clear Water Bay, Kowloon, Hong Kong, 999077, P. R. China

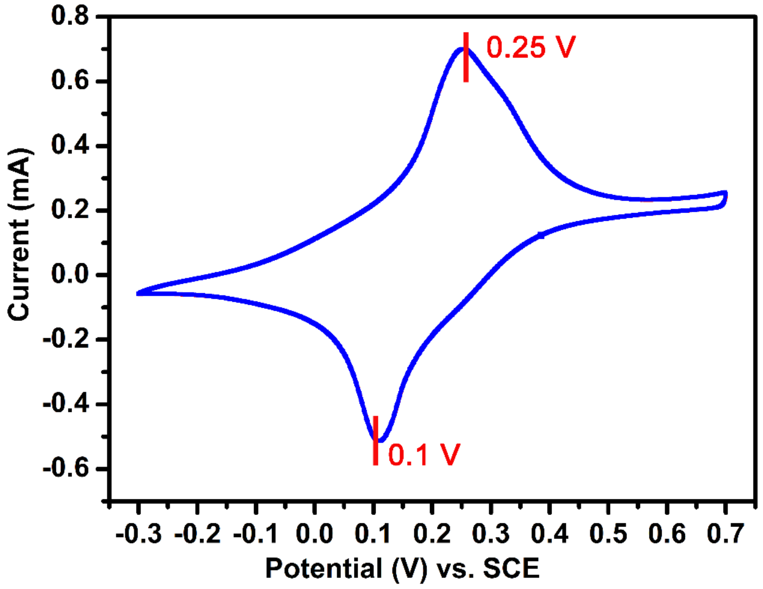

Figure S1. Cyclic voltammetry curves acquired at a scanning rate of 10 mV s^{-1} from the carbon cloth substrate.

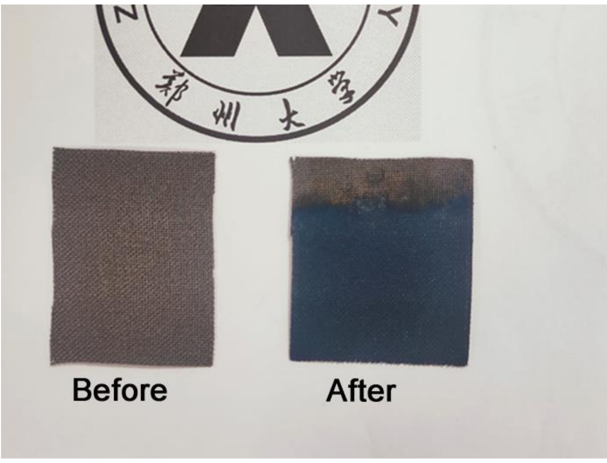

Figure S2. Optical image of the carbon cloth before and after electrodeposited the KFeHCF nanoparticles.

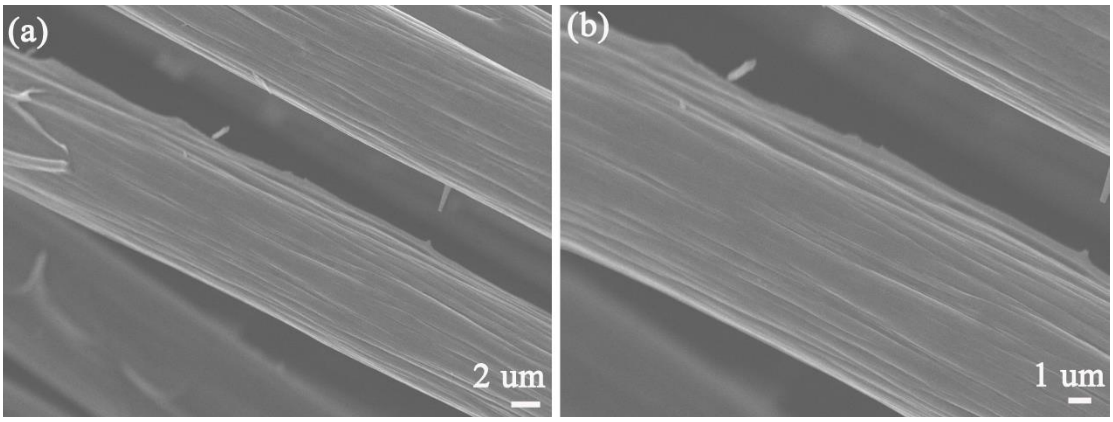

Figure S3. (a, b) SEM images of the carbon cloth.

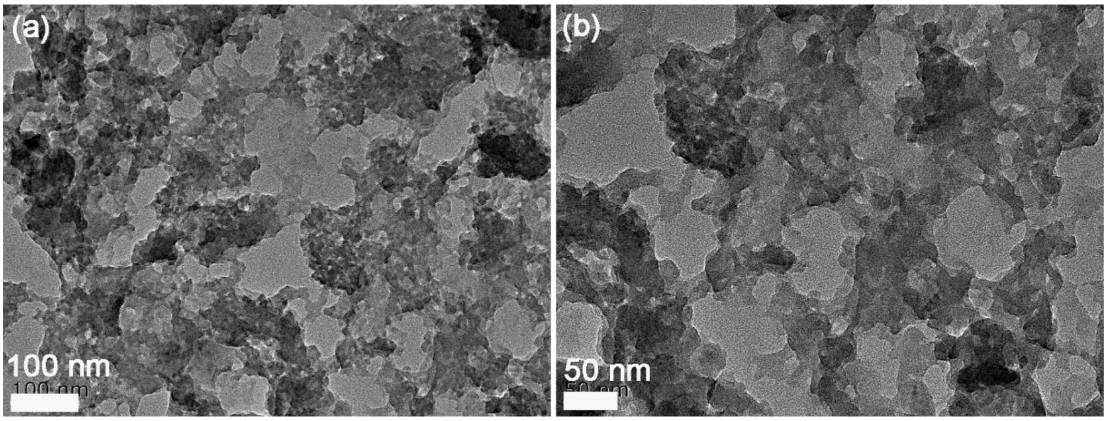

Figure S4. (a, b) TEM images of the as-electrodeposited KFeHCF nanoparticles stripped off from the carbon cloth.

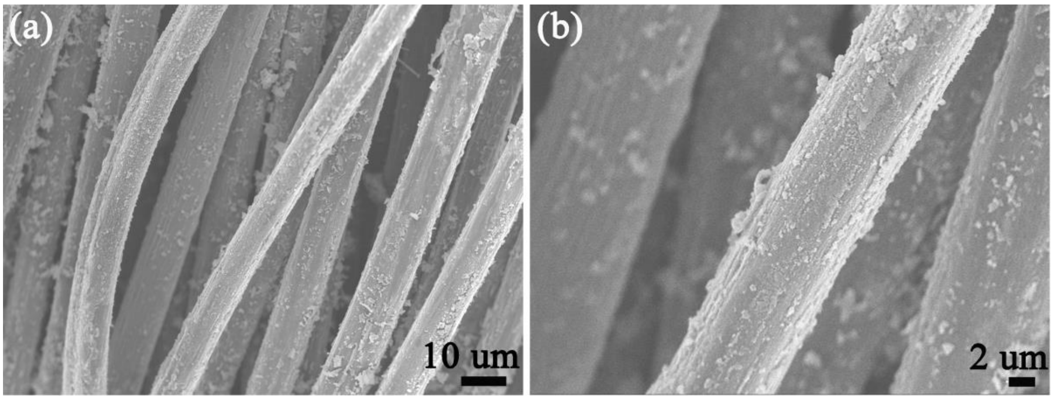

Figure S5. (a, b) SEM images of the KFeHCF/CC after twisting and bending 20 cycles.

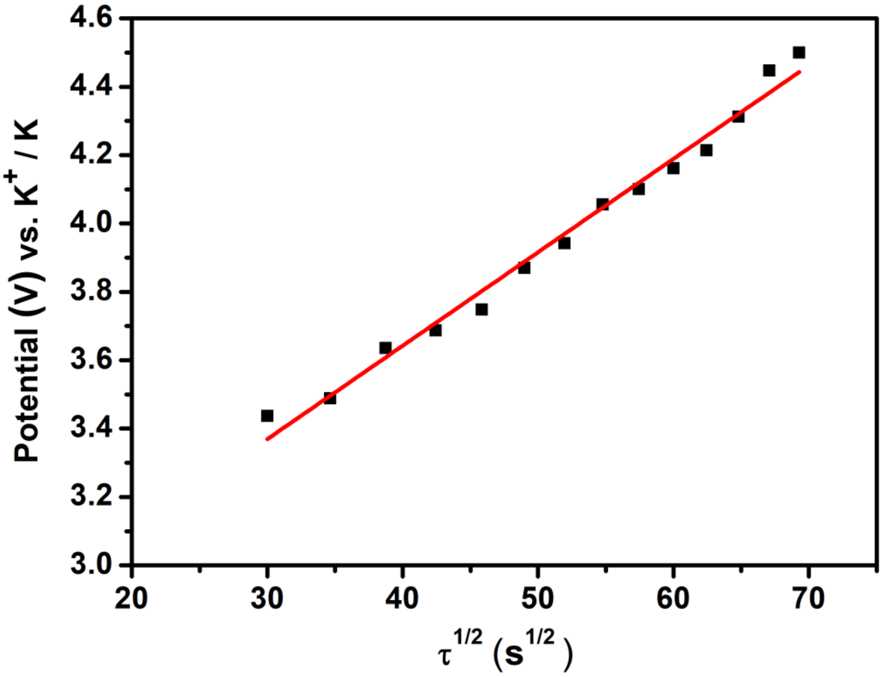

Figure S6. Linear relationship of Potential vs. $\tau^{1/2}$ in GITT of KFeHCF/CC for KIBs.

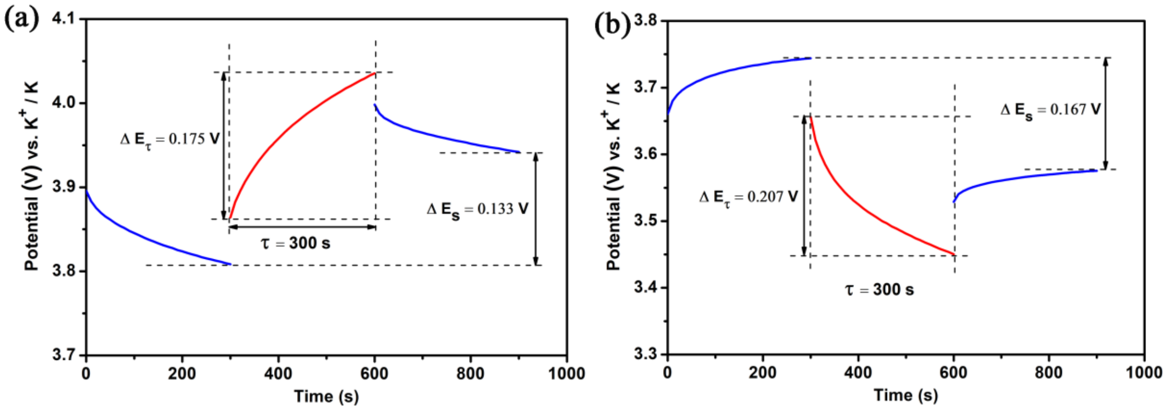

Figure S7. The time versus voltage curves for a single titration during (a) the charging process and (b) the discharging process, respectivly.

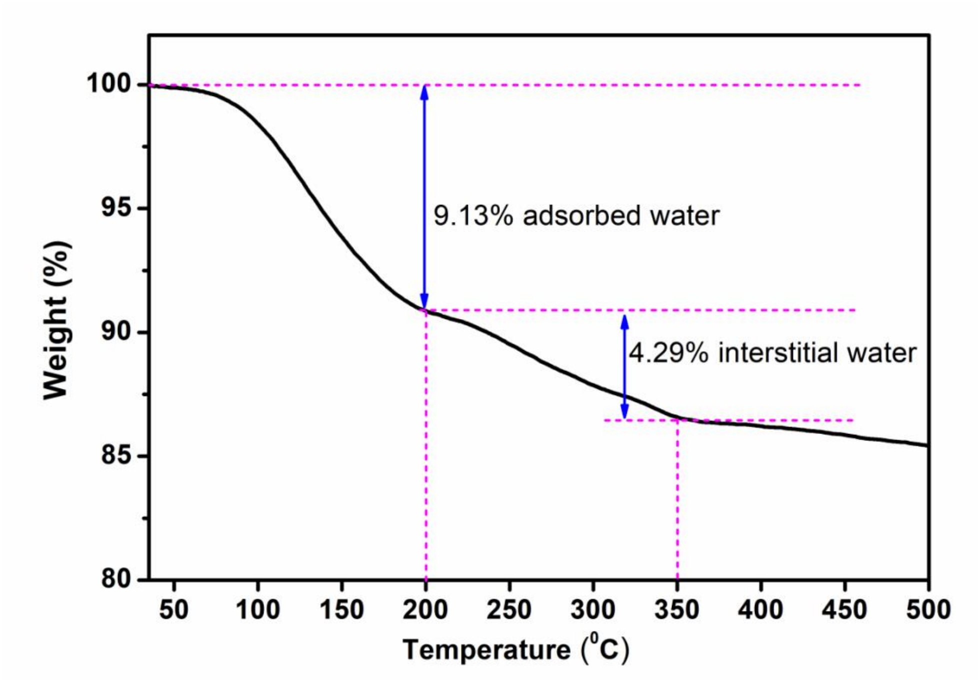

Figure S8. TGA curve of the KFeHCF sample at a scan rate of 10 ^{0}C min^{-1} in N_2 atomosphere.
